# *In situ* macromolecular crystallography using microbeams

**DOI:** 10.1107/S0907444912006749

**Published:** 2012-04-17

**Authors:** Danny Axford, Robin L. Owen, Jun Aishima, James Foadi, Ann W. Morgan, James I. Robinson, Joanne E. Nettleship, Raymond J. Owens, Isabel Moraes, Elizabeth E. Fry, Jonathan M. Grimes, Karl Harlos, Abhay Kotecha, Jingshan Ren, Geoff Sutton, Thomas S. Walter, David I. Stuart, Gwyndaf Evans

**Affiliations:** a Life Science Division, Diamond Light Source, Harwell Science and Innovation Campus, Didcot, Oxfordshire OX11 0DE, England; b Membrane Protein Laboratory, Imperial College, London SW7 2AZ, England; c NIHR–Leeds Musculoskeletal Biomedical Research Unit and Leeds Institute of Molecular Medicine, University of Leeds, Leeds LS9 7FT, England; d OPPF-UK, Research Complex at Harwell, Rutherford Appleton Laboratory R92, Didcot, Oxfordshire OX11 0DE, England; e Division of Structural Biology, Wellcome Trust Centre of Human Genetics, University of Oxford, Roosevelt Drive, Oxford OX3 7BN, England

**Keywords:** *in situ* diffraction, microfocus, microbeams, high throughput, room temperature, viruses, membrane proteins, synchrotron radiation

## Abstract

Despite significant progress in high-throughput methods in macromolecular crystallography, the production of diffraction-quality crystals remains a major bottleneck. By recording diffraction *in situ* from crystals in their crystallization plates at room temperature, a number of problems associated with crystal handling and cryoprotection can be side-stepped. Using a dedicated goniometer installed on the microfocus macromolecular crystallography beamline I24 at Diamond Light Source, crystals have been studied *in situ* with an intense and flexible microfocus beam, allowing weakly diffracting samples to be assessed without a manual crystal-handling step but with good signal to noise, despite the background scatter from the plate. A number of case studies are reported: the structure solution of bovine enterovirus 2, crystallization screening of membrane proteins and complexes, and structure solution from crystallization hits produced *via* a high-throughput pipeline. These demonstrate the potential for *in situ* data collection and structure solution with microbeams.

## Abstract 2

A sample environment for mounting crystallization trays has been developed on the microfocus beamline I24 at Diamond Light Source. The technical developments and several case studies are described.

## 1. Introduction   

The mounting of crystals in loops or on meshes and their cryoprotection remains a manual and often painstaking process in macromolecular crystallography (MX), in contrast to the successful automation of many other steps in the sequence-to-structure pipeline. Some years ago, the measurement of diffraction data from crystals at room temperature within the crystallization trays used for their growth was shown to be feasible on a bending-magnet beamline (Jacquamet *et al.*, 2004 ▸). Jacquamet and coworkers demonstrated that a large defocused X-ray beam of ∼1 mm in size could be used to provide useful information about the diffraction quality of crystals within a drop. In certain cases, diffraction data sets could also be measured from crystals of a few hundred micrometres in size. More recently, ligand-soak experiments have been performed *in situ* (le Maire *et al.*, 2011 ▸), and at the Swiss Light Source a crystallization facility now adjoins a beamline capable of *in situ* data collection (Bingel-Erlenmeyer *et al.*, 2011 ▸). These implementations use a robotic arm to support and manipulate crystallization plates in order to perform measurements. The positional resolution and repeatability of these robotic arms limit their usefulness for crystal and X-ray beam sizes much less than 20 µm, sizes that are typical for a microfocus beamline such as I24.

Many of the most challenging targets in structural biology yield small and weakly diffracting crystals, and the ability to record *in situ* diffraction from such crystals is essential if the method is to have an impact on these problems. In this manuscript, we highlight three areas where *in situ* measurements with microbeams can have a particularly strong impact: diffraction screening of crystallization experiments in membrane-protein crystallography, data collection from virus crystals and structure solution to complement high-throughput crystallization pipelines. The advent of crystallization facilities such as the Membrane Protein Laboratory (MPL; http://www.diamond.ac.uk/Home/MPL.html) and the Oxford Protein Production Facility (OPPF-UK; http://www.oppf.ox.ac.uk/OPPF/) at Diamond Light Source is attractive because the production of diffraction-quality crystals remains a significant bottleneck (Geerlof *et al.*, 2006 ▸) and bringing together crystallization and X-ray diffraction screening may assist in crystal optimization and structure solution. In recent years, the use of robots to prepare high-density (usually 96-well SBS-format) plates using nanolitre volumes in each drop has lead to a rapid increase in the number of crystallization drops, and hence potential crystals, handled in many laboratories. The typically small size of the crystals produced from nanodrop crystallization (Santarsiero *et al.*, 2002 ▸) means that the magnitude of diffuse X-ray scatter from both the tray and the mother liquor surrounding the crystal is comparable to, or greater than, that of Bragg diffraction from the crystal, so that potential data could be obscured by noise. Thus, although laboratory-based plate-screening X-ray sources are available (for example, the PX Scanner; Agilent Technologies; http://www.chem.agilent.com), these systems are limited in the information that they can provide owing to modest flux coupled with a relatively large (250 µm) beam size. As a result, experimenters continue to mount and cryocool putative crystals individually for screening either on a home source or at a synchrotron beamline. The ability to collect high-quality data directly from crystallization drops *in situ* raises the prospect of providing immediate unambiguous feedback on the nature of newly formed crystals.

The manipulation and cryocooling of membrane-protein, virus and other large complex crystals is a challenging and time-consuming process. For membrane-protein crystals grown in mesophase this is even trickier, since visualization of the crystals is difficult. Solubilization and stabilization are key to the success of membrane-protein crystallization; therefore, the use of detergent plays an important role. However, the presence of detergent micelles covering most of the hydrophobic surface of the protein reduces protein–protein contacts, resulting in crystal lattices with high solvent content. Consequently, membrane-protein crystals initially tend to be small, extremely fragile, sensitive to temperature variation, poorly ordered (often having a high mosaic spread) and very sensitive to radiation damage. To improve crystal quality, many crystallization conditions have to be explored, increasing the number of crystals produced. Handling a large number of small fragile crystals can be problematic and often leads to false negatives, which complicate crystal optimization. *In situ* X-ray data collection of membrane-protein crystals effectively eliminates many of these challenges. Similarly, and out of necessity, virus crystallography is often conducted using room-temperature samples owing to the deleterious effect of cryocooling on crystal mosaic spread, leading to the overlap of diffraction spots from adjacent layers of the reciprocal lattice. In addition, the strain introduced into the crystal frequently eliminates the high-resolution diffraction. This problem is most severe when the unit cell is very large. Collecting diffraction data *in situ* avoids this problem and maintains the integrity of crystals which are often very fragile and do not respond well to traditional handling methods. An additional consideration in virus crystallography is the very real disease-security problem inherent in collecting data from open loops or grids. This may be overcome by mounting crystals in sealed thin-walled capillaries (Fry *et al.*, 1993 ▸). However, this is time-consuming and impractical for the most delicate crystals, so that crystal growth directly in capillaries has sometimes been used (Cockburn *et al.*, 2003 ▸). *In situ* analysis in sealed plates provides a safe alternative for pathogenic samples.

*In situ* data collection has not yet emerged as a routine method. This is principally owing to the radiation-sensitivity of protein crystals at room temperature, where the crystal lifetime is typically two orders of magnitude less than at 100 K (Southworth-Davies *et al.*, 2008 ▸), but also reflects a lack of goniometry optimized for handling crystallization plates with high precision, the rotational restrictions and the unsuitability of most crystallization plates for diffraction measurements. However, reflecting the current interest in the field, several dedicated plates are now commercially available, such as the Topaz 1.96 DC (Fluidigm; http://www.fluidigm.com), Crystal­Harp (SWISSCI; http://www.swissci.com) and CrystalQuick X (Greiner Bio-One; http://www.greinerbioone.com), or have recently been described (Soliman *et al.*, 2011 ▸; Kisselman *et al.*, 2011 ▸). Desirable properties of the plate design include a low physical profile maximizing the achievable rotation range, a wide solid opening angle for exiting diffraction onto the detector and thin well bases to minimize both X-ray scatter and refraction effects when rotating samples. A comparison of plate types is reported in Bingel-Erlenmeyer *et al.* (2011 ▸). The CrystalQuick X plate has a reduced thickness of the base of each well: reduced from ∼1 mm to 250–300 µm. Angled walls to each reservoir allow a potential 80° angular range for data collection. Topaz 1.96 DC chips have a base thickness of 200 µm and an even greater angular range since the design does not require reservoir wells.

Here, we describe a high-precision goniometer that is capable of holding crystallization plates mounted on the MX beamline I24 at Diamond Light Source and presenting each of the crystallization drops to the microfocus X-ray beam. We also present some of the results obtained whilst bringing this device into routine use.

## 2. Materials and methods   

### 2.1. Experimental apparatus   

**Figure 1:**
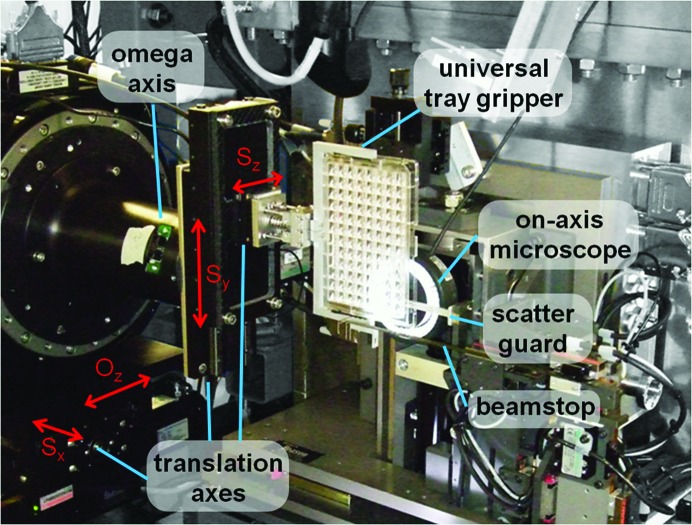
Photograph of the I24 sample-environment setup used for *in situ* data collection. The translation axes enable any position within the crystallization plate to be centred onto the ω-rotation axes. The *O*_*z*_ axis enables the rotation axis to be translated along the direction of the beam and positioned at the focal point of the on-axis viewing system. Thus, movement along this axis can correct for changes in the focal length of the viewing system owing to the plate-refraction effects described in §[Sec sec2.2]2.2. The translations *S*_*x*_, *S*_*y*_ and *S*_*z*_, with ranges of travel of 80, 150 and 12 mm, respectively, are used to bring a crystal to the centre of rotation and into the X-ray beam. The scatter guard, beamstop and on-axis microscope are used as part of the setup for standard data collections. The gripper is capable of holding most SBS-format plates, including glass LCP plates.

The *in situ* screening setup utilizes much of the existing sample-environment apparatus at I24. This includes the sample ω-rotation stage, the on-axis sample-viewing microscope, scatter guards and beam stop (Fig. 1 ▸). The typical I24 beam size of <10 µm and its ability to routinely record data from frozen crystals of <10 µm in size (Ji *et al.*, 2010 ▸) led us to develop a solution for holding and positioning plates in the X-­ray beam using high-precision linear stages mounted on the existing high-precision ω axis (Aerotech ABRS-250MP), ensuring that high-quality diffraction data can be measured from small crystals in plates.

The standard I24 sample-centring stage is replaced by one capable of holding most SBS-format crystallization plates in portrait orientation. This stage is capable of manipulating plates so that crystals in any drop can be accurately positioned on the rotation axis. The vertical axis (Heason/Nanomotion) provides 150 mm of travel, while the axis parallel to the X-ray beam (Attocube) provides 12 mm of travel. Movement of the plate horizontally perpendicular to the beam is accomplished using a long travel stage beneath the ω axis. The mechanical resolution of positioning is better than 0.1 µm and the repeatability is better than 0.5 µm. The limited angular range used by the plate goniometer means that it does not suffer greatly from errors arising from changes in gravitational load across the axes as they rotate. In comparison, the regular goniometer head (Maatel) has a resolution of <0.25 µm and a repeatability of <2 µm across a full 360° rotation.

As far as possible, the amount of equipment around the sample stage has been minimized, in particular above and below the rotation axis. The remaining spatial limitations result in an available rotation range of ±25°: effort was not made to increase the range further, as beyond angles of ∼25° refractive effects make the visualization of crystals difficult. Plates are currently manually transferred to the goniometer, but automated transfer from a ‘plate hotel’ at the beamline using the CATS robot is under development.

### 2.2. Handling of crystallization-plate optical effects   

For conventional single-crystal diffraction experiments at Diamond the sample is typically viewed using an on-axis microscope and aligned optically with the centre of rotation in two orthogonal directions (the rotation axis is pre-aligned so that it intersects the X-ray beam at the focal plane of the sample-viewing optics). This ensures that the sample stays centred on the rotation axis at all possible angles. This simple procedure becomes more challenging for microcrystals, where optical refraction effects in the material surrounding the crystals can shift the apparent position of the crystal(s). Diffraction methods are routinely used in these cases to locate and centre samples (Song *et al.*, 2007 ▸; Aishima *et al.*, 2010 ▸), but the principle of centring in two orthogonal directions remains the same.

The precise alignment of crystal samples with the rotation axis when they are held within a crystallization plate cannot be performed in the same way. It is not possible to perform alignment with the axis of rotation by turning through 90° owing to the size and shape of the crystallization plate. One centring option is to rely on the narrow depth of the focal plane of the on-axis microscope: crystals can be aligned with the rotation axis by bringing them into focus. However, we have observed that in addition to adding to background X-ray scatter, the plate shifts the focal plane away from the viewing optics. If a crystal is to remain within the beam during rotation, the rotation axis must be shifted to correct for this optical effect. This is essential for *in situ* microcrystallography because a 2° rotation can result in as much as a 14 µm lateral motion (full details are given in the Supplementary Material^1^).

**Figure 2:**
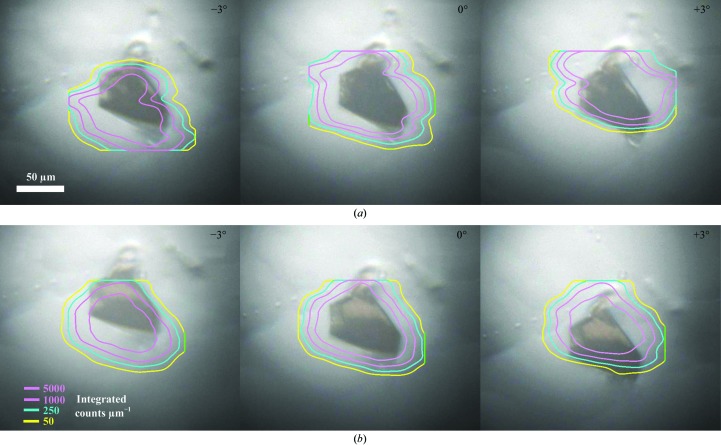
Sample positions within a crystallization plate as determined using X-ray diffraction grid scans (the contour plots represent crystal X-ray diffraction strength) and using visible light through the on-axis microscope, which is subject to refraction effects that systematically shift the apparent crystal location. (*a*) and (*b*) show series of three images recorded with ω equal to −3°, 0° and 3°. The images in (*a*) were recorded with the sample-rotation axis position in the nominal OAV focal plane, whereas the images in (*b*) were recorded with the axis of rotation translated 400 µm downstream into the new focal plane of the OAV–plate combination. Note that in (*a*) the crystal appears visually to remain centred when rotated, but the diffraction data confirm that it is in fact drifting off-axis. In (*b*) the crystal appears to translate vertically when rotated, but X-ray diffraction confirms that is correctly centred on the axis of rotation.

Tray-induced optical effects can be illustrated through use of the diffraction grid scan tool available at I24. The case of a standard-format Greiner CrystalQuick SW crystallization plate is shown in Fig. 2 ▸. In Fig. 2 ▸(*a*) the sample is not coincident with the axis of rotation, but appears stationary from the point of view of the observer when the plate is rotated. When the tray is rotated by ±3° the grid scan shows that the crystal position shifts by 20 µm. When a sample is coincident with the axis of rotation, as confirmed by the grid scan (Fig. 2 ▸ *b*), it appears through the sample-viewing optics to be translating vertically when rotated.

Knowledge of the appropriate offset for a given plate can be included in the experimental setup, allowing samples to be aligned accurately when the plate is perpendicular to the viewing optics. However, two further complications have been observed. Firstly, crystals do not always rest on the plate surface but may be suspended within the crystallization drop: the likelihood of this increases when the plate is held vertically for data collection. Offset of the crystal from the plate results in further displacement of the focal plane (Supplementary Fig. S1^1^) and this must also be accounted for. Secondly, some crystallization plates have curved well bottoms, resulting in optical displacement in both the horizontal and the vertical directions (an example is shown in Fig. 6*b*). Correction for this requires knowledge of the exact position of the well and its curvature relative to the viewing optics. This correction is not currently made at I24 and alignment must be carried out using diffraction grid scanning, with the deleterious side effect of irradiating crystals before data collection. Use of the grid scan has also proven to be useful for the detection of protein crystals within opaque lipidic phases which can hinder visual alignment. In practice, it has been found that offset of the rotation axis is sufficient for alignment of crystals in most cases and this correction has proven to be essential for the successful alignment of microcrystals.

We have found that the position and the shape of drops within a plate are stable on mounting in a vertical orientation. On occasion, we observe crystals moving under gravity and coming to rest at the bottom of a drop. The likelihood of movement as a function of sample size and shape has not yet been quantified. It is possible to collect data from crystals that have moved, with no apparent effect on data quality.

### 2.3. Sample preparation   

Crystallization experiments for bovine enterovirus 2 (BEV2; a virus that requires no disease precautions and that was grown and purified in the Department of Microbiology, University of Leeds by Professor D. Rowlands) were set up in 96-well Greiner CrystalQuick SW and CrystalQuick X plates using a Cartesian robot (approximately 10 µl BEV2 at a concentration of 7 mg ml^−1^ per plate; Walter *et al.*, 2005 ▸). The sitting drops were formed from 100 nl virus solution plus 100 nl precipitant in 1:1, 2:1 and 1:2 ratios. The bar-coded trays were sealed with Greiner VIEWseal tapes and stored at 293.5 K. Crystals of bipyramidal morphology (space group *F*23, unit-cell parameter *a* ≃ 437 Å) grew in many conditions in both plate types.

A protein–DNA–peptide complex was purified at Cancer Research UK (Lincoln’s Inn Fields, London). Initial crystallization trials with different-sized DNA oligomers were carried out at the London site using MRC-format (SWISSCI) plates with a protein concentration of 10 mg ml^−1^. Many small crystals grew over a two-week period. Further crystallization trials subsequent to initial *in situ* testing used Greiner Crystal­Quick X plates and were set up at the MPL using a Mosquito robot (TTP LabTech).

Crystals of three membrane proteins were generated at the MPL. A bacterial membrane-anchor cytochrome protein was expressed and purified at the Instituto De Tecnologia Quimica e Biologica (ITQB) in Portugal. Crystallization experiments were set up at the MPL in 96-well Greiner CrystalQuick X plates. Triangular bipyramid crystals grew within a week in 300 nl sitting drops. A bacterial membrane protein was expressed and purified as two separate domains at the ITQB, concentrated to ∼30 mg ml^−1^ and then transported to the MPL for crystal growth and optimization as a complex of the two domains in MRC-format (SWISSCI) plates. Crystals appeared in 1 d and grew over 7 d into hexagonal plates. A human G-protein-coupled receptor (GPCR) was expressed in Sf9 cells at Evotec (UK). Purification and crystallization trials were performed at the MPL in lipid cubic phase (LCP) and small needle-shaped crystals appeared within one week.

Two polymorphic variants of FcγRIIIA, 7040 and 7041, were expressed, purified and crystallized at OPPF-UK (Research Complex at Harwell). 7040 differs from 7041 by a single amino-acid substitution: F158V (Ravetch & Perussia, 1989 ▸). Full details of sample preparation and structure determination will be published elsewhere. Crystallization followed similar protocols to those for BEV2, using Greiner CrystalQuick SW plates. 7040 crystals were typically ∼30 × 30 × 30 µm in size; they were of multiple morphologies and were often fused together in clusters. Crystals of polymorph 7041 grew under identical crystallization conditions and were of similar size and morphology.

### 2.4. Data collection   

**Figure 3:**
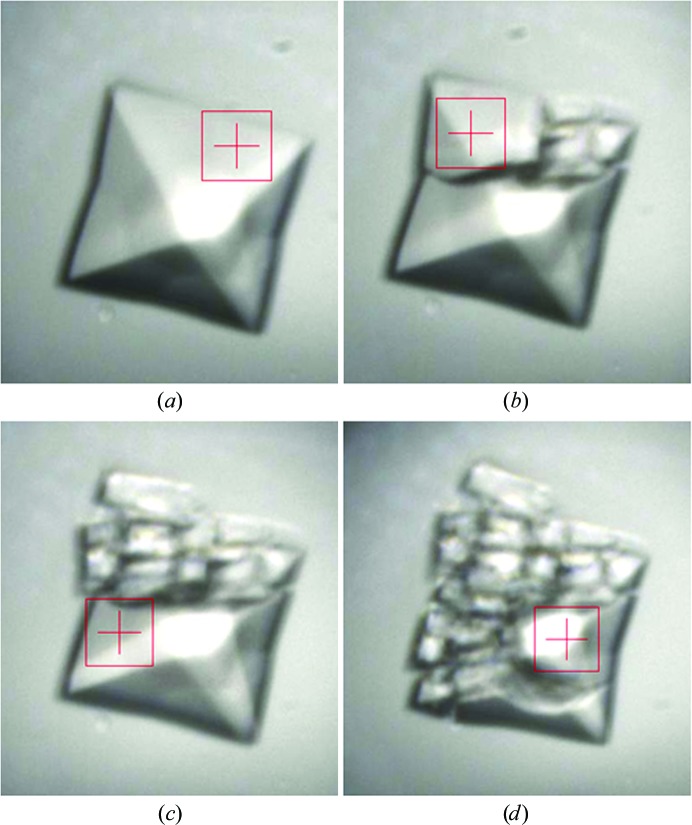
Four images of a BEV2 crystal during data collection. (*a*) Prior to any exposure. (*b*) After a 0.5 s exposure. (*c*) After a second 0.5 s exposure. (*d*) After a third 0.5 s exposure. The beam cross-section of 20 × 20 µm is shown.

For all experiments, plates were manually mounted in the plate holder and initially centred on a drop containing crystals. The rotation axis was offset according to the tray type to allow accurate crystal centring. For larger crystals it was often possible to collect data from more than one position, as shown in Fig. 3 ▸. For logistical efficiency in locating crystals, optical images of all drops containing crystals obtained from the crystal imagers (Daniel *et al.*, 2011 ▸) were viewed alongside the image from the beamline camera. All data were collected on a Pilatus 6MF detector operating in shutterless mode at 294 K, the ambient temperature of the beamline.

For BEV2, individual frames comprised either 0.1° or 0.05° and exposure times close to the minimum allowed by the frame rate of the detector (typically 0.1 s or less), as long as this regime led to data of sufficient strength for relatively routine processing. Under the regime described here the crystal lifetime was typically ∼0.4 s; the effects of radiation damage were clearly visible (Fig. 3 ▸). For this reason, data were generally collected at 12.8 keV (chosen to allow the U21 undulator to be operated at close to its minimum gap, maximizing the photon flux to >10^12^ photons s^−1^ at the sample in a beam size of 20 × 20 µm). CrystalQuick X plates gave superior data to standard Greiner CrystalQuick SW plates, enabling data extending to 2.1 Å resolution to be collected (Supplementary Material^1^). Background scatter was reduced by around a factor of 2.5 on moving to the X-ray-specific plate type. The CrystalQuick X plates gave a comparable background to a conventional loop at low (>10 Å) resolution and high (<2.5 Å) resolution, but have a peak in background around 5 Å that is approximately three times that of a loop. The data presented were gathered from a single CrystalQuick X plate in a single session on I24.

For FcγRIIIA, diffraction data were recorded from 66 crystals of 7040 using 12.68 keV X-rays, a beam size of 20 × 20 µm and a photon flux attenuated to ∼5 × 10^11^ photons s^−1^ at the sample. A partial data set consisted of approximately ten 0.5° oscillation images each of 0.1 s duration. Using the same experimental strategy, diffraction data were also collected from 120 crystals of 7041.

### 2.5. Data processing   

Data frames of BEV2 were indexed and integrated with *HKL*-2000. The mosaic spread of each data wedge was refined using the first three frames and was then fixed during integration (this procedure is stable but not ideal, since the mosaic spread can increase during irradiation). Data wedges were scaled and merged using *SCALEPACK*, with the mosaic spread fixed and partially recorded reflections from contiguous frames merged.

In the case of FcγRIIIA, data were integrated using *XDS* (Kabsch, 2010 ▸). Sequences of blank images or extremely weak diffraction arising from radiation-damage-induced decay or the crystal rotating out of the beam were rejected manually before integration with *XDS*.

## 3. Results   

### 3.1. Data collection from BEV2 crystals   

Data-collection and processing statistics are given in Table 1 ▸. Data from 28 crystals of ∼50–60 µm in size were merged to yield a data set more than 80% complete to 2.5 Å resolution.

**Figure 4:**
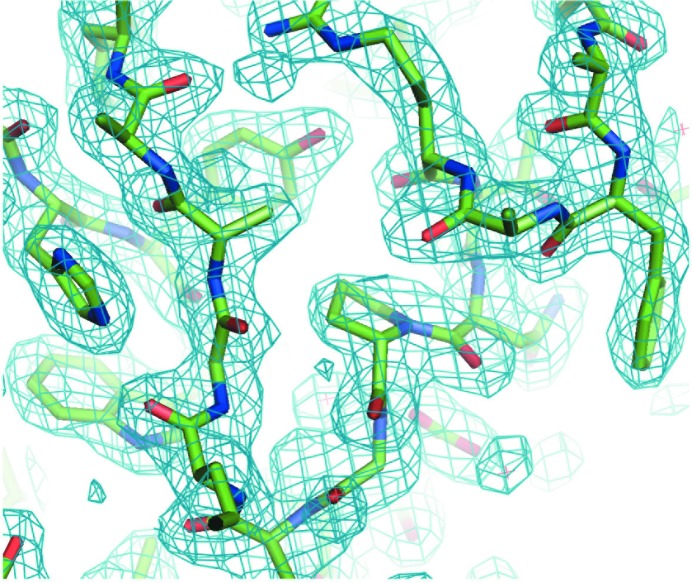
A section of the 2.1 Å resolution 2*F*_o_ − *F*_c_ electron-density map of BEV2 contoured at 1.5σ.

The structure was solved by molecular replacement using the BEV1 structure (PDB entry 1bev; Smyth *et al.*, 1995 ▸), averaged (*GAP*; D. I. Stuart, J. M. Grimes & J. Diprose, unpublished work) and refined (*CNS*; Brünger *et al.*, 1998 ▸) using strict noncrystallographic symmetry constraints. Sample electron density is shown in Fig. 4 ▸; details of the structure solution and the structure will be published elsewhere.

### 3.2. Comparison of data from a multi-component complex under cryogenic conditions and *in situ*   

**Figure 5:**
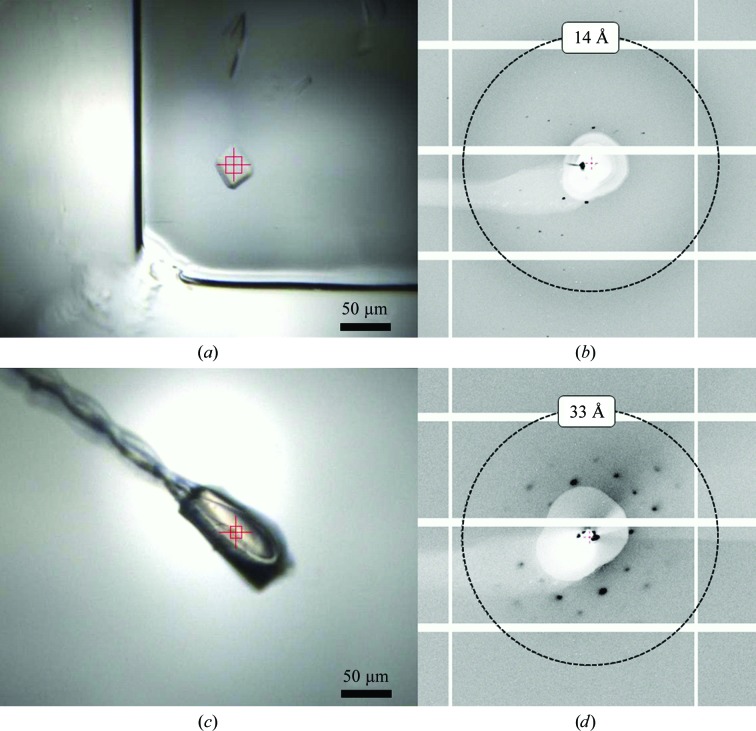
Comparison of *in situ* and frozen data collection from crystals of a protein–DNA complex. View of a crystal in a drop within a crystallization plate (*a*) and the diffraction obtained from this sample (*b*). Spots were observed to 10 Å resolution. A crystal grown in identical conditions was mounted in a fibre loop and cryocooled to 100 K (*c*). The resulting diffraction extended no further than 35 Å resolution (*d*) from the cryocooled crystal despite it being a larger size. *In situ* data collection allows a much more accurate assessment of crystallization conditions during the process of crystallization optimization.

Diffraction data obtained *in situ* extended to a higher resolution than could be observed from the same samples under cryogenic conditions. Fig. 5 ▸ shows the example of two crystals of a 48 kDa protein–DNA–peptide complex obtained from identical crystallization conditions. The respective diffraction images in Figs. 5 ▸(*b*) and 5 ▸(*d*) show that despite their smaller size, diffraction from *in situ* crystals extends further (9 *versus* 36 Å) with a lower mosaic spread. In this example, the improved diffraction results allowed differences to be resolved in the quality of crystals from the different conditions, whereas all crystals yielded a similar diffraction limit of around 36 Å at 100 K. The information from the *in situ* analysis was used to select the most promising conditions for further optimization.

### 3.3. Membrane-protein crystal screening   

**Figure 6:**
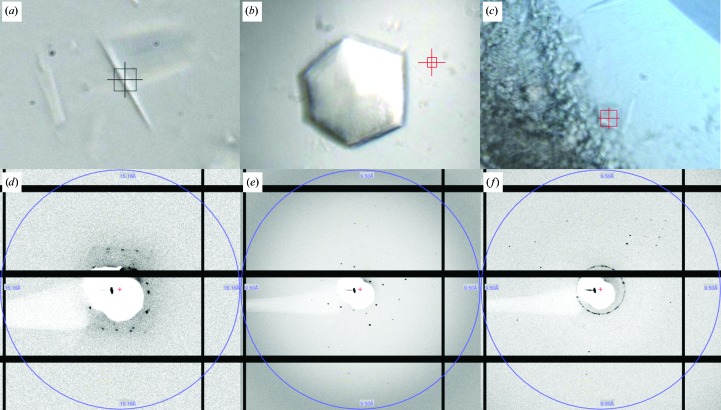
On-axis microscope images of crystal hits and *in situ* diffraction patterns from three example membrane proteins. (*a*, *d*) A G-protein-coupled receptor protein crystallized in lipid cubic phase. (*b*, *e*) A complex of two domains of a bacterial membrane protein (the apparent misalignment of the beam centre and crystal in this case is a consequence of large refraction effects induced by the use of curved bottomed wells). (*c*, *f*) A bacterial membrane-anchor cytochrome protein. In (*a*), (*b*) and (*c*) the square box and cross-hair represent the beam size and position, respectively. In each case the size was 10 × 10 µm.

Results from the three examples taken from the MPL membrane-protein crystallization optimization program are shown in Fig. 6 ▸. In each case, *in situ* diffraction screening facilitated a significant saving of time and manual effort by avoiding manual handling. Additionally, more samples could be tested *in situ* since typically only one crystal could be removed from each drop before any remaining crystals melted. In Fig. 6 ▸(*a*) the crystal is in an LCP-specific plate which would require the breaking of a glass cover slip for the crystal to be mounted in a loop. The associated diffraction image in Fig. 6 ▸(*d*), despite attenuation from the glass cover slip, confirms that the crystal is protein and allows a unit-cell dimension to be measured. In Fig. 6 ▸(*b*), despite the refractive effects of a curved bottomed plate, following crystal location using the grid scan diffraction could be measured to 9.5 Å resolution. In Fig. 6 ▸(*c*), crystals contained in Greiner Crystal­Quick X plates and with a largest dimension of <20 µm demonstrated diffraction to 10 Å resolution. In both Figs. 6 ▸(*e*) and 6 ▸(*f*) the extent of measurable diffraction allowed a rapid ranking of different crystallization conditions.

### 3.4. Structure solution from high-throughput pipeline initial crystal hits   

**Figure 7:**
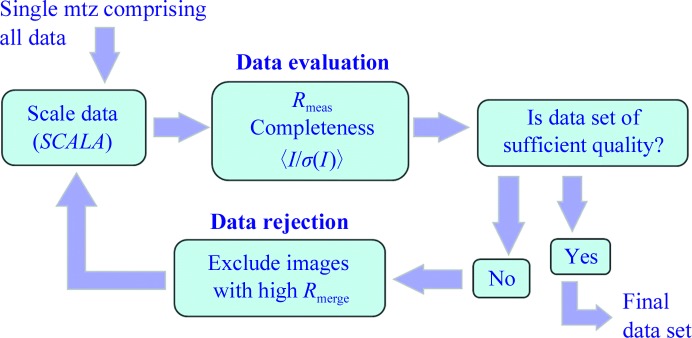
Strategy used for obtaining a data set from a large number of thin wedges (0.5–1.5°) of diffraction data.

For FcγRIIIA, data from all diffraction wedges were merged using the *CCP*4 program *POINTLESS* (Evans, 2011 ▸). Owing to the short lifetime of each crystal, a robust strategy capable of producing a final data set of acceptable quality and completeness was devised (Fig. 7 ▸). *SCALA* (Evans, 2006 ▸) was run iteratively until an optimized data set was obtained. In early iterations the scale factors had to be fixed to allow all weak data to be included. Outlier images (those with the largest batch *R*_merge_) were successively excluded. After 6–8 cycles the data could be scaled using default parameters. After 18 (7040 polymorph) and 12 (7041 polymorph) iterations the metrics *I*/σ(*I*) and *R*_meas_ showed only minor improvement as further images were excluded from scaling, while the completeness began to fall. An optimal data set was chosen based on comparison of *I*/σ(*I*), *R*_meas_ and completeness. As a rule of thumb and a basis for automating this process we anticipate removing a certain percentage of images per cycle until the proportionate decrease in completeness exceeds the proportionate decrease in *R*_meas_. However, formalizing thresholds for these statistical measures will need to be appropriate for the aim of the experiment. The optimized data set was then used for molecular replacement with *MOLREP* (Vagin & Teplyakov, 2010 ▸) using the 3.2 Å resolution crystal structure of the human IgG1 Fc fragment–Fcγ receptor III complex (PDB entry 1e4j; Sondermann *et al.*, 2000 ▸) as a search model. The solution was refined using *REFMAC* (Murshudov *et al.*, 2011 ▸), *PHENIX* (Adams *et al.*, 2010 ▸) and *Coot* (Emsley *et al.*, 2010 ▸). The resulting electron density was easily interpretable (Supplementary Fig. S3^1^). Scaling and refinement statistics are given in Table 2 ▸.

## 4. Discussion   

The ability to obtain information on the diffraction quality of crystallization hits *in situ* has proved particularly valuable in overcoming the challenges of membrane-protein crystallization and cocrystallizations to produce complexes, in which the effective search space for optimum conditions is greatly increased with each additional component. Indeed, *in situ* characterization is now a routine part of the optimization pipeline for the MPL, with regular access on a roughly monthly basis subject to the operations schedule as part of the beamline user time. To date, it has been applied to over a dozen MPL projects. This method of data collection typically allows the experimenter to record improved diffraction data, reduces the risk of false negatives, enables a faster progression of crystallization optimization and eliminates crystal manipulation. What the early experiments have shown strikingly is that in addition to this role in screening crystallization hits, *in situ* data collection can yield data sets of high quality and may offer an attractive path to routine structure determination for certain potentially challenging structures.

By eliminating crystal handling, plate-based data collection allows modern synchrotron beamlines to take advantage of the inherently low mosaic spread of many virus crystals and provides a safe platform for the analysis of pathogenic particles. The current generation of purpose-designed crystallization plates (such as the Greiner CrystalQuick X and the DC version of Fluidigm chips) allow data to be collected with modest background and good signal to noise. The net result is that even at this early stage of development (where there is still great scope for improvement) it is possible to routinely collect sufficient data to solve a virus structure at room temperature and at high resolution from crystals of 50 µm or less in size from a single 96-well plate with a few hours of beamtime, as demonstrated by the experiment recorded in Table 1 ▸. The method described here is now in routine use and has led to over a dozen virus structures (data not shown) from crystals belonging to rhombohedral, cubic and orthorhombic space groups.

In the case of FcγRIIIA, using a relatively simple iterative scaling strategy it was possible to merge weak diffraction data from a large number of small data sets of less than 4° each. This protocol is quite different to current practice for macromolecular crystallography, in which single crystals are normally removed manually from the crystallization drop and (either before or after removal from the drop) cryoprotected before cooling to 100 K. Advantages of the *in situ* protocol are as follows. Firstly, it becomes possible to complete high-throughput gene-to-structure pipelines with automated diffraction data collection, circumventing the, as yet, almost exclusively manual crystal-mounting stage. Secondly, the method of merging many thin wedges of weak data has direct applications in several challenging situations, including micro-crystallography, where the expected lifetime of a crystal can be less than ten images even at 100 K (Evans *et al.*, 2011 ▸), forcing experimenters to merge data from an increasingly large number of crystals for structure solution (see, for example, Ji *et al.*, 2012 ▸).

Subsequent to the iterative scaling step for 7040 and 7041, structure solution and refinement progressed as normal. The large attrition rate for images (<20% contributing to the final merged data sets) can be attributed in part to the refractive effect described in §[Sec sec2.2]2.2, which was still being characterized during this experiment. Images obtained with the crystal partially out of the beam were discarded during these steps. Although the scaling statistics are inferior to those expected from single crystals at 100 K, the electron density was readily interpretable and novel features such as bound *N*-acetyl-d-­glucosamine could be easily observed.

### 4.1. General outlook   

The introduction of tray hotels at beamlines such as I24, coupled with the availability of dedicated tray-capable gonio­meters, will make high-throughput diffraction screening of microcrystals a reality. Furthermore, it is clear from our initial results that advances in automated data processing will make the analysis of diffraction data from such crystals a routine crystallographic method, enabling not only crystal characterization but also structure solution from crystals held in trays at room temperature. These hardware and software developments are essential if what is currently a niche method, used by a small but growing number of groups, is to be transformed into one which is widely used.

The ongoing development of crystallization plates is also key to the development of *in situ* MX. While the simplest approach to reducing the scatter of plates is to make wells of ever thinner materials, the choice of material is also important if scatter in the resolution ranges of interest is to be minimized. For weakly diffracting samples even the scattering power of the sealing tape should be considered for optimal measurements. Well shape is also important: a curved well base may be preferable for liquid handling, but it introduces unwanted refraction effects making crystal alignment difficult.

In addition to the established issues of microcrystal sample centring, there is an immediate need for specific software tools to aid data collection *in situ*. For example, to achieve efficient thoughput, a robust method to feed positional coordinates of crystals recorded from laboratory crystallization systems directly into the beamline goniometer position would greatly speed up sample location and centring for data collection and could even lead to a completely automated system. When collecting data from multiple samples within one drop it is essential to be able to efficiently track which crystals and which subvolumes of larger samples have been exposed (at least in the case of samples not suffering visible radiation damage). Additionally, the automation of data integration and merging for multi-crystal data collection will save the experimenter considerable time. Indeed, near-real-time assessments of the data quality and completeness will provide quantitative feedback on when to conclude a data collection and move on to the next project.

## Supplementary Material

Supporting information file. DOI: 10.1107/S0907444912006749/tz5002sup1.pdf

## Figures and Tables

**Table 1:** Scaling statistics for BEV2 based on *in situ* data collection using a Greiner CrystalQuick X plate on microfocus beamline I24 at Diamond Light Source, UK Values in parentheses are for the outermost resolution shell. Four to six useful images were collected from each position.

Space group	*F*23
Unit-cell parameters (Å)	*a* = *b* = *c* = 436.6
No. of crystals/No. of exposed positions	28/76
Resolution range (Å)	50–2.1 (2.18–2.10)
〈*I*/σ(*I*)〉	4.3 (1.2)
*R*_merge_	0.196 (0.404)
*R*_meas_	Not calculated
Completeness (%)	65.8 (17.2)†
No. of unique observations	260688 (6779)
Multiplicity	2.3 (1.2)

† >80.0% to 2.5 Å resolution.

**Table 2:** Scaling and refinement statistics for the 7040 and 7041 polymorphs of FcγRIIIA Values in parentheses are for the outermost resolution shell.

	7040	7041
Space group	*P*6_1_22	*P*6_1_22
Unit-cell parameters (Å)	*a* = *b* = 65.34, *c* = 178.31	*a* = *b* = 60.6, *c* = 213.8
No. of crystals used	44	72
Resolution range (Å)	57–2.4 (2.53–2.40)	57–2.4 (2.53–2.40)
Initial No. of images integrated	527	514
No. of images forming final data set	87	97
No. of scaling cycles required	18	12
〈*I*/σ(*I*)〉	5.3 (1.9)	4.7 (1.6)
*R*_merge_	0.180 (0.783)	0.231 (0.878)
*R*_meas_	0.201 (0.870)	0.256 (0.970)
Completeness (%)	95.5 (97.0)	98.3 (99.0)
No. of unique observations	8941 (1290)	9613 (1362)
Multiplicity	4.6 (4.8)	5.1 (5.4)

## Footnotes

1 Supplementary material has been deposited in the IUCr electronic archive (Reference: TZ5002). Services for accessing this material are described at the back of the journal.
