# Supplementary material for: In situ macromolecular crystallography using microbeams
Source: Acta Crystallogr D Biol Crystallogr. 2012 Apr 17;68(Pt 5):592–600. doi: 10.1107/S0907444912006749 (PMC4791750; doi:10.1107/S0907444912006749)
Supplement: Supplementary file 1 [file d-68-00592-sup1.pdf]

Person to contact for this submission:
[please select a primary contact author using the Author details tool]

Supplementary information:

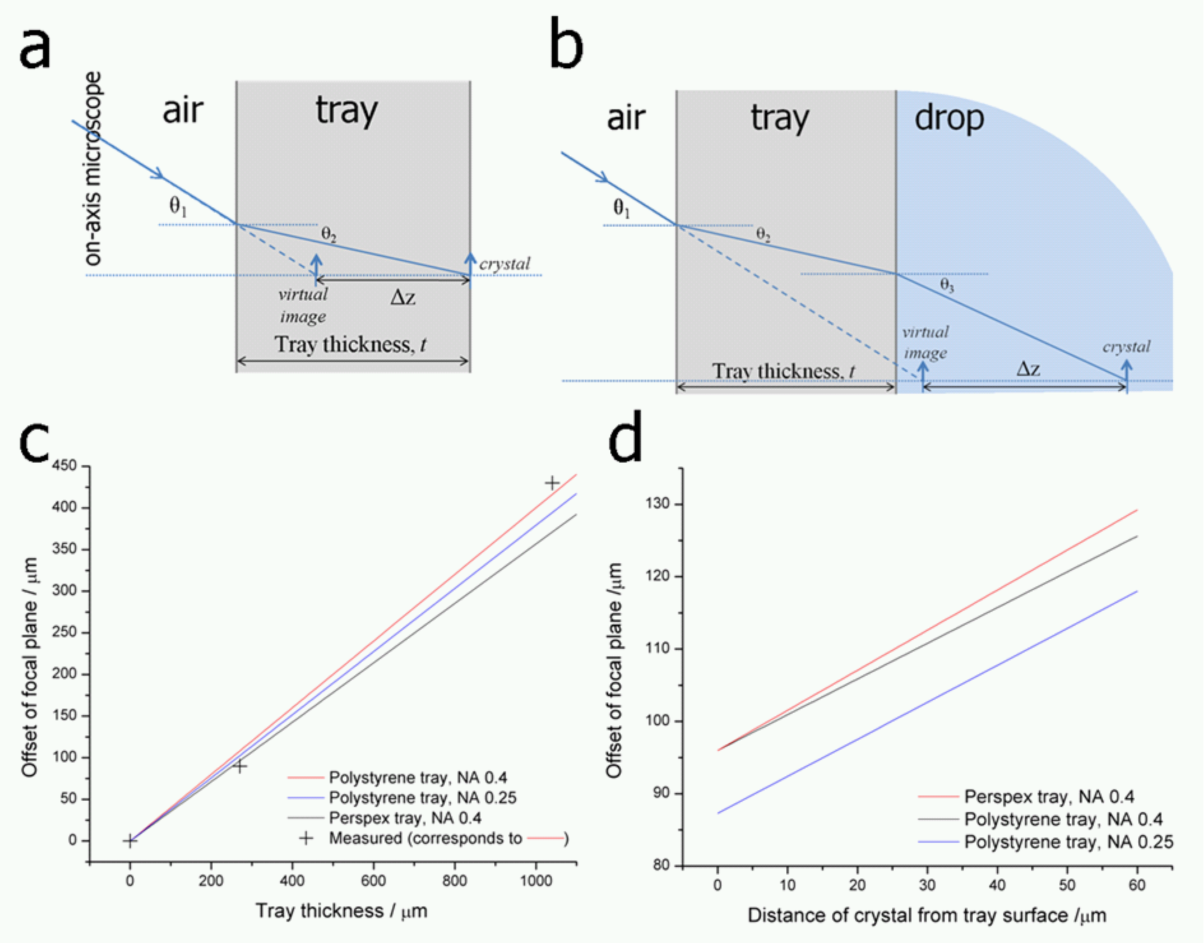

Figure 1 Ray diagrams showing offset of focal plane due to a crystallisation tray (a) and a crystallisation tray plus drop (b). Offset of focal plane shown due to finite thickness of tray (b). Shown are offsets measured using two types of tray. Offset of focal plane for crystals some distances from the tray surface (d).In all cases Δz is the offset of the focal plane; the rotation axis must be offset by this amount if the crystal is to remain centred within the beam during data collection.

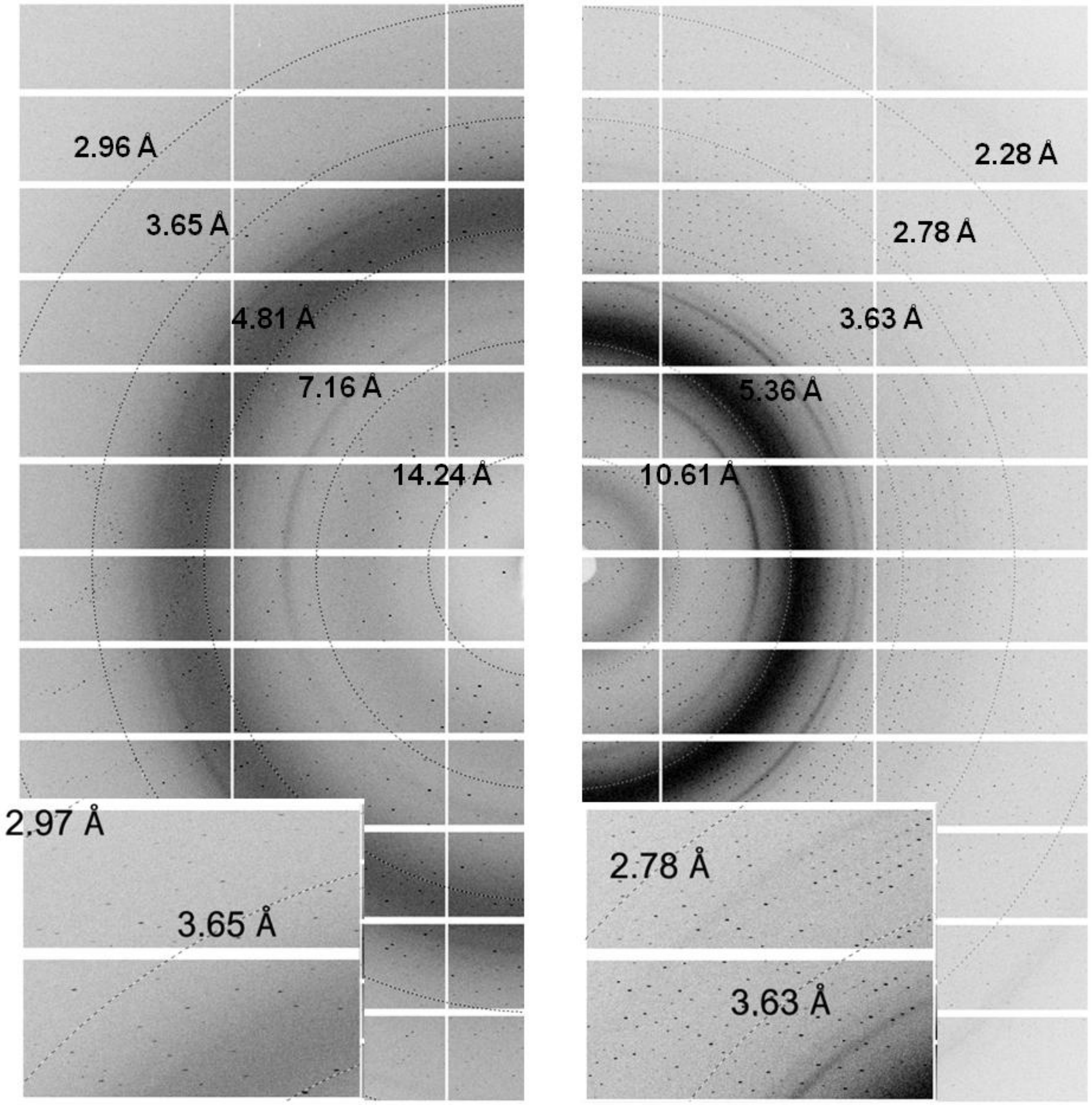

Figure 2 *In situ* diffraction images of BEV2 crystals using (a) Greiner 'CrystalQuick SW' and (b) Greiner 'CrystalQuick X' crystallisation plates. Both images were collected at Diamond Light Source beamline I24 with Pilatus 6MF detector at wavelength λ=0.97780 Å, ~10^{12} ph/s, exposure time 0.1 sec and oscillation 0.1°. Resolution at edge of detector is 2.97 Å in (a) and 2.28 Å in (b). The ring current was 200 mA. The crystal to detector distance was 645 mm in (a) and 480 mm in (b). The average number of counts per pixel at 3.5 Å resolution was 28.5 (a) and 20.4 (b). The diffracting volume in each case was within 10%. Taking into account the difference in crystal to detector distance this corresponds to a reduction in background by a factor of 2.5 at 3.5 Å resolution for the 'CrystalQuick X' plate.

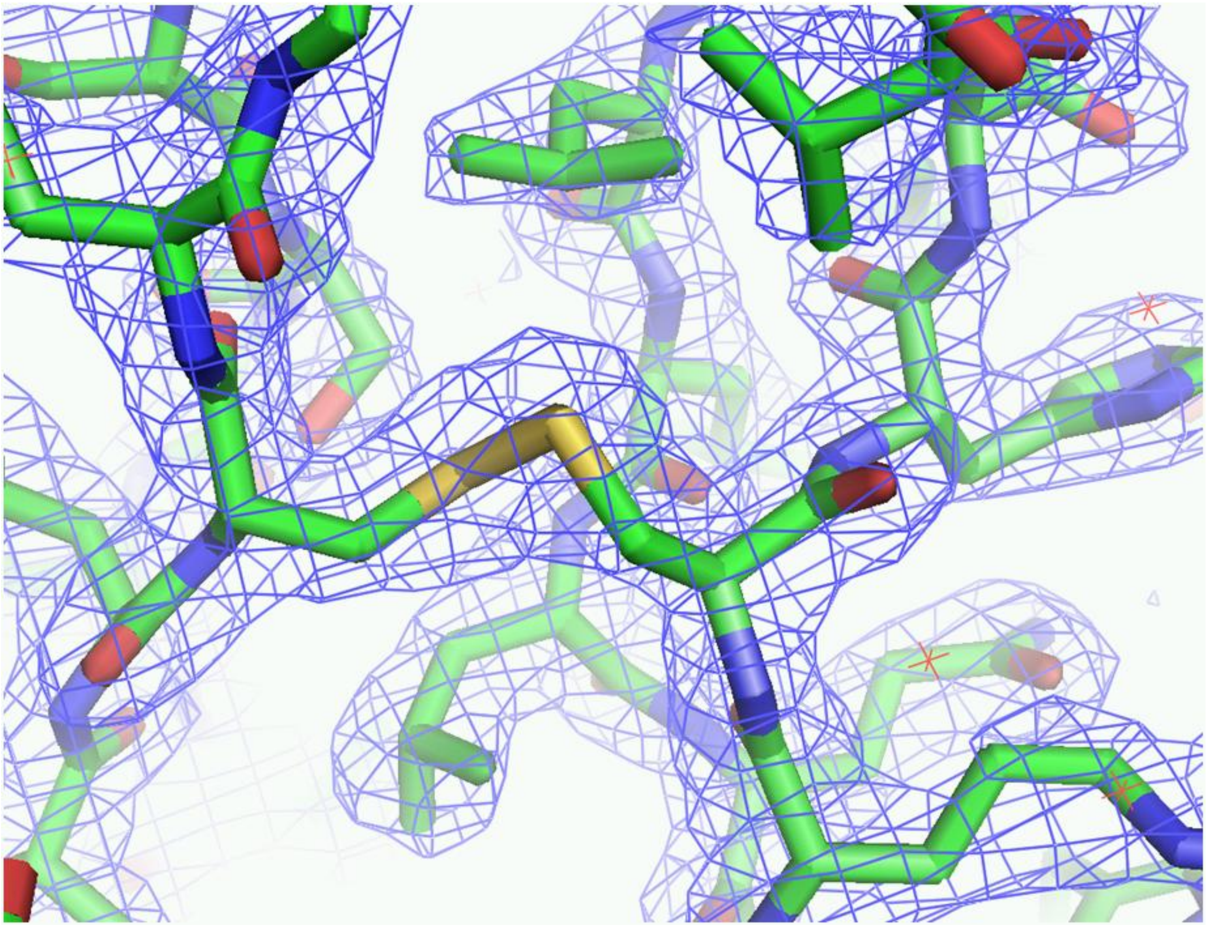

Figure 3 Showing electron density, contoured at 1.5σ, around a disulphide bridge in the 7041 polymorph of FcγRIIIA.
